# A Sensitive Carbon Monoxide Sensor Based on Photoacoustic Spectroscopy with a 2.3 μm Mid-Infrared High-Power Laser and Enhanced Gas Absorption

**DOI:** 10.3390/s19143202

**Published:** 2019-07-20

**Authors:** Shunda Qiao, Yufei Ma, Ying He, Xin Yu, Zhonghua Zhang, Frank K. Tittel

**Affiliations:** 1 National Key Laboratory of Science and Technology on Tunable Laser, Harbin Institute of Technology, Harbin 150001, China; 2 Department of Electrical and Computer Engineering, Rice University, 6100 Main Street, Houston, TX 77005, USA

**Keywords:** photoacoustic spectroscopy (PAS), gas sensor, PA cell, mid-infrared laser

## Abstract

A photoacoustic spectroscopy (PAS)-based carbon monoxide (CO) gas sensor with a high-power laser and an enhanced gas absorption was demonstrated. The light source was a distributed feedback (DFB), continuous wave (CW) diode laser with a high output power of ~8 mW to give a strong excitation. The target gas received optical absorption enhanced two times by using a right-angle prism reflecting the laser beam. In order to reduce the noise from the background, wavelength modulation spectroscopy (WMS) and second-harmonic detection techniques were used. The modulation frequency and modulation depth were optimized theoretically and experimentally. Water vapor was added in the PAS sensor system to increase the vibrational–translational (V–T) relaxation rate of the CO molecule, which resulted in an ~8 times signal enhancement compared with the using of a dry CO/N_2_ gas mixture. The amplitude of the *2f* signal had a 1.52-fold improvement compared to the one with only one time absorption. The experimental results showed that such a sensor had an excellent linear response to the optical power and gas concentration. At 1 s integration time, a minimum detection limit (MDL) for CO detection of 9.8 ppm was achieved. The long-term stability of the sensor system was evaluated with an Allan deviation analysis. When the integration time was 1100 s, the MDL improved to be 530 ppb. The detection performance of such a PAS-based CO sensor can be further improved when a laser with a higher output power and increasing optical absorption times is used.

## 1. Introduction

Carbon monoxide (CO) is a colorless and odorless gas. In daily life, CO is produced with incomplete combustion when fossil fuels and natural gas are used. CO combines easily with hemoglobin in the blood which can form carboxyl hemoglobin and make the hemoglobin lose the ability and function of carrying oxygen. This result will cause tissue asphyxia and death in severe cases [1]. It was also verified that long-term exposure to low concentration CO will be harmful for the health of people [2]. Furthermore, the CO from human breath with a concentration at the ppm level can be used to monitor the disease [3,4]. In addition, CO is an increasingly serious source of air pollution. CO reacts with hydroxyl (OH) in the air causing global warming [5]. Therefore, it is important to develop a sensitive CO gas sensor.

The photoacoustic (PA) effect was first reported by Alexander Graham Bell in 1880. Bell found that optically absorbing materials shined with scattered sunlight produced an audible sound. Then Kreuzer reported that laser-induced PA generation could be used to detect ultra-trace gas constituents at concentrations of the part per billion (ppb) level [6]. Subsequently, many applications based on the PA effect were demonstrated. Photoacoustic spectroscopy (PAS) was an important technique which was an application of the PA effect. When a laser beam was directed on the target gas, the gas molecules absorb the optical radiation and are excited. These excited molecules can relax to the ground state via a non-radiative process which produce localized heating. Heating made the volume of the target gas expand. When the laser beam was turned off, the volume of the gas would shrink. Therefore, it can be seen that when the target gas was excited with a modulated laser beam, the volume exhibits a periodic change and a localized pressure wave is produced. An acoustic wave was generated along with the pressure wave which can be detected by a microphone [7]. PAS is widely used in the detection of trace gases and has many advantages, such as a high detection sensitivity, a fast response, and a wide dynamic range.

The PA cell is an important part of the trace gas sensor based on PAS. The generated PAS signal is detected inside the PA cell. The geometry of the PA cell influences the performance of the sensor system significantly [8,9]. In order to obtain a higher PAS signal, the modulation frequency of the laser should be optimized. When the modulation frequency is equal to the resonant frequency of the PA cell, the acoustic wave produced by the target gas forms a standing wave in the PA cell, which means that the PAS signal can be amplified. Therefore, a resonant PA cell is widely used in PAS-based trace gas sensors [10,11]. The intensity of the PAS signal has a relationship with the absorbed radiation by the target gas. When the optical power does not reach the nonlinear absorption regime of the target gas, the gas absorption coefficient is constant. The amount of absorbed power has a linear relationship with the laser power. If more optical power is absorbed by the target gas, the intensity of the acoustic signal will be higher, which means that the gas sensor has a higher detection sensitivity. Therefore, a laser source with a higher output power can be used to improve the optical absorption of the gas. Many PAS-based sensors with a high-power laser source have been already demonstrated [12,13,14]. Furthermore, the detection sensitivity of PAS-based sensors could also be improved with the increase of absorption times [15,16,17].

In this paper, a PAS-based CO gas sensor with a high optical power laser source and an enhanced absorption with double pass was demonstrated. A distributed feedback (DFB), continuous wave (CW) diode laser emitting at 2.3 μm with high output power of ~8 mW was used as the excitation source. A right-angle prism was used to reflect the laser beam to parallelly invert and was passed through the PA cell two times. Water vapor acting as a catalyst was added in the PAS sensor system to increase the vibrational–translational (V–T) relaxation rate of the CO molecule. A resonant PA cell with two buffers designed to reduce the noise from gas flow was also employed. A condenser microphone was used to detect the PAS signal. To reduce the noise from the background, wavelength modulation spectroscopy (WMS) and second-harmonic detection techniques were used. The modulation frequency and modulation depth were optimized theoretically and experimentally. With the optimum parameters of the sensor system and an integration of 1 s, a minimum detection limit (MDL) for CO detection of 9.8 ppm was achieved. The long-term stability of such a sensor system was evaluated with an Allan deviation analysis. 

## 2. Experimental Setup

### 2.1. Absorption Line Selection

**Figure 1:**
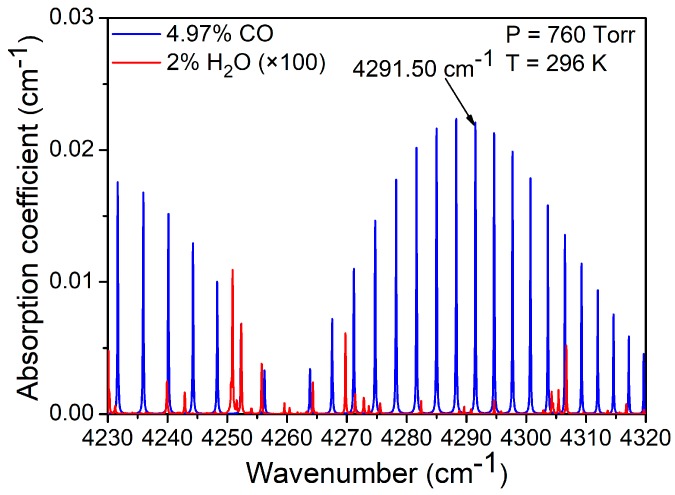
CO absorption lines in the 2.3 μm first-overtone absorption band.

A gas molecule has different absorption lines with different absorption intensity. According to the HITRAN 2012 database [18], the CO absorption lines in the 2.3 μm first-overtone absorption band are shown in Figure 1. It can be seen that an absorption line at the wavelength of 2330.19 nm (4291.50 cm^−1^) is one of a series of strong absorption lines and has no interference with water vapor (H_2_O). Therefore, this absorption line was chosen in our experiments.

### 2.2. Laser Source

**Figure 2:**
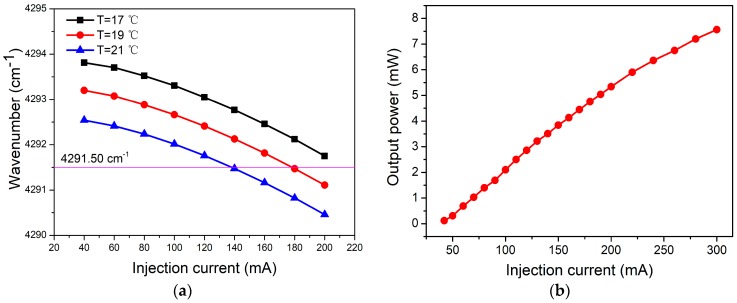
(**a**) The wavenumber of a continuous wave (CW), distributed feedback (DFB), 2.3 μm laser as a function of injection current at different temperatures. (**b**) The output power of a CW, DFB, 2.3 μm laser as a function of injection current.

Because of the CO absorption line located at the wavelength of 2330.19 nm (4291.50 cm^−1^), a CW, DFB laser with emission in this range was used. The laser was mounted in a 14-pin butterfly package including a thermoelectric controller (TEC). The wavelength of laser can be tuned by changing the injection current and the temperature of the TEC to cover the CO absorption line. The wavenumber of the laser at different temperatures with injection current tuning is shown in Figure 2a. It can be seen that at a certain temperature, the wavenumber of the laser source has a nearly linear response to the injection laser. The tuning coefficient of the wavenumber was ~0.013 cm^−1^/A. When the temperature of the TEC was set to 19 °C, the output power of the laser as a function of the injection current is shown in Figure 2b. The maximum optical power was 7.82 mW when the injection current was 300 mA. The tuning coefficient of the optical power was ~0.03 mW/A.

### 2.3. Sensor Configuration

**Figure 3:**
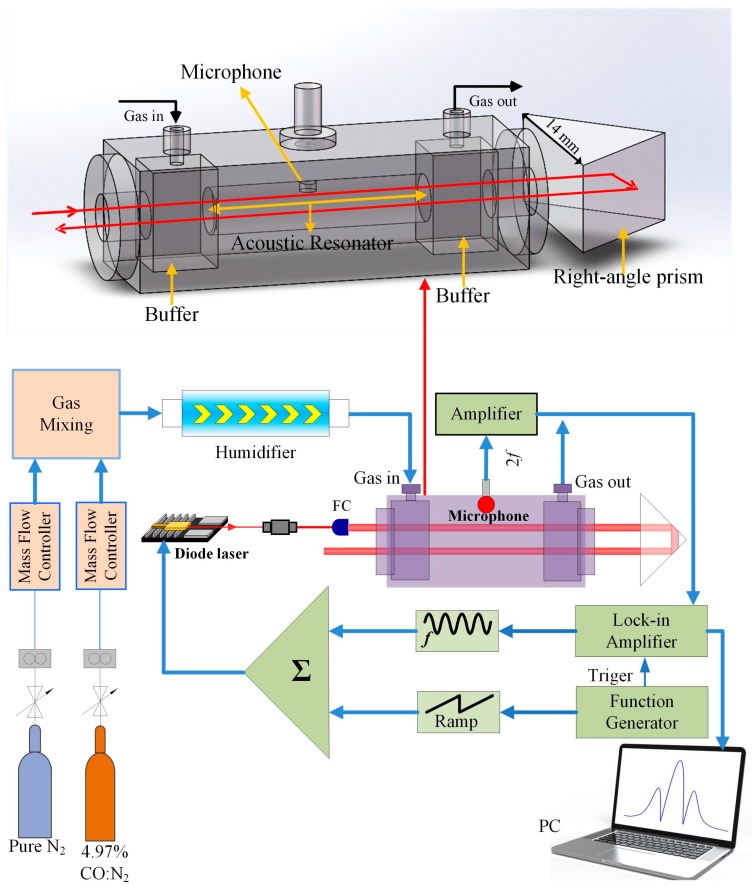
Schematic configuration of the photoacoustic spectroscopy (PAS)-based sensor system. FC: fiber collimator.

The schematic of the PAS-based CO gas sensor system is shown in Figure 3. The laser beam emitting from the CW, DFB, 2.3 μm diode laser was collimated with a fiber collimator (FC). Then the laser beam passed through a resonant PA cell. The PA cell has a central cylinder tube with a radius and length of 5 and 100 mm, respectively, which was used as the acoustic resonator. Two buffers were set at both sides of the central cylinder to reduce the noise from the gas flow. A microphone was set inside the PA cell. The total length of the PA cell was 200 mm. In order to increase the absorption, a right-angle prism made from fused quartz with a side length of 14 mm was used to reflect the laser beam and make the laser parallel inverted and pass through the PA cell again. A humidifier (water vapor) was added in the PAS sensor system to increase the vibrational–translational (V–T) relaxation rate of the CO molecule. The WMS and second-harmonic detection techniques were used to reduce the noise from the background. The function generator produced a sawtooth wave and a high level signal. The sawtooth wave was used to scan the wavelength of the laser to cover the CO absorption line and the high level signal was used to trigger the lock-in amplifier to demodulate the second harmonic. The lock-in amplifier provided a sine wave to modulate the laser source. In order to obtain the optimum PAS signal, the frequency of the sine wave should be half of the resonant frequency of the PA cell. The integration time of the sensor system was set to 1 s.

## 3. Theoretical Optimization of the WMS

**Figure 4:**
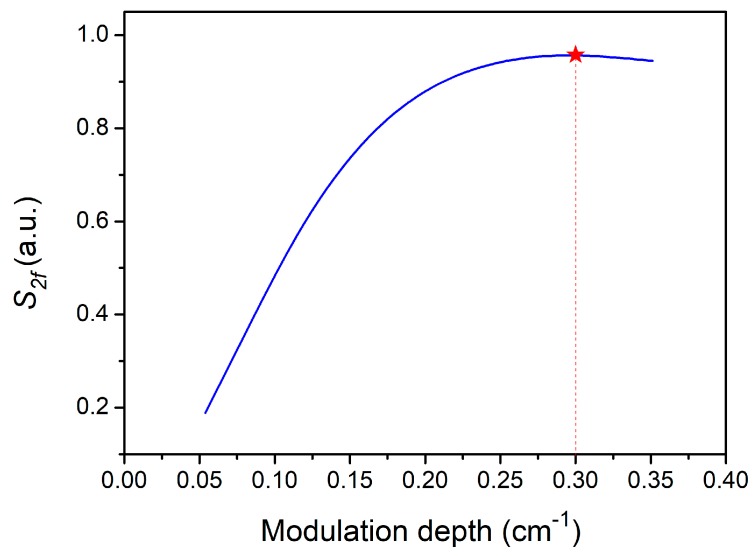
Second harmonic signal S*_2f_* as a function of modulation depth.

WMS and second-harmonic detection techniques are widely used in trace gas sensor systems. The two techniques can reduce the noise from the background and improve the performance of the sensor. A sine wave with a high frequency was adopted to modulate the laser wavelength and the harmonic can be detected with a lock-in amplifier. For the odd harmonics, the intensity of the harmonic component at the peak of the gas absorption line was zero, so that it cannot be used to invert the gas concentration. However, the even harmonics have an extremum at the peak of the gas absorption line, and with the increase of the harmonic times, the intensity of the harmonic component decreased quickly. Therefore, the second harmonic was the best selection to be used as the detected signal. In WMS, $H_{0}(\bar{x})$ and $H_{n}(\bar{x})$ are the harmonic coefficients for the harmonic signals and they are expressed as Equations (1) and (2) [19,20]:

(1) $$H_{0}(\bar{x})=\frac{1}{\pi }\int_{0}^{\pi }\frac{1}{1+{\left(\bar{x}-M\cos(\theta )\right)}^{2}}d\theta ,$$

(2) $$H_{n}(\bar{x})=\frac{2}{\pi }\int_{0}^{\pi }\frac{\cos(n\theta )}{1+{\left(\bar{x}-M\cos(\theta )\right)}^{2}}d\theta ,$$

where wavenumber $\bar{x}=(\bar{\nu }-\nu_{0})/\gamma$ is the non-dimensional wavenumber deviation from the line center $\nu_{0}$, $\bar{\nu }$ is the central wavenumber of the laser, *γ* is the absorption line width, $M=\delta_{\nu }/\gamma$ is the modulation depth coefficient, $\delta_{\nu }\;$is the magnitude of modulation wavenumber. The second harmonic signal is used to retrieve gas concentrations when applying the WMS technique and the signal S*_2f_* in PAS is given by Equation (3) [21,22]:

(3) $$\begin{array}{ll} S_{2f}(\nu ) & =kC_{cell}{\left[\alpha (\nu )P(\nu )\right]}_{2f}\\ & =kC_{cell}\alpha_{0}P_{0}[(1+P_{\Omega }\bar{x})H_{2}\cos(2\omega_{m}t+2\varphi )] \end{array}$$

where $C_{cell}\;$is a constant for the sensor system, *k* is a conversion constant of the system, *α*_0_ and *P_0_* are the absorption coefficient and laser power at the gas absorption line center, respectively, $P_{\Omega }$ is the laser power coefficient for scanning (slow ramp) at a frequency φ, and $f=\omega_{m}/2\pi$ is the modulation frequency. The second harmonic acoustic coefficient in the bracket of Equation (3) can be used to perform a theoretical simulation for S*_2f_* and determine the optimum modulation depth. The CO absorption line located at 4291.50 cm^−1^ was selected. According to the HITRAN 2012 database, the line width *γ* for the absorption line located at 4291.50 cm^−1^ is 0.135 cm^−1^. The calculated results are shown in Figure 4. From Figure 4, it can be seen that the maximum signal amplitude is obtained when the modulation depth $\delta_{\nu }$ was chosen to be 0.3 cm^−1^.

## 4. Results and Discussion

**Figure 5:**
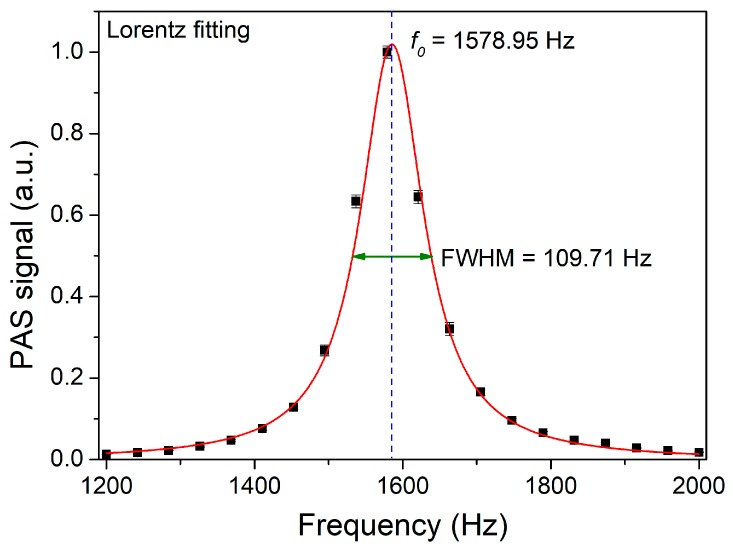
Frequency response of the photoacoustic cell. FWHM: full width at half maximum.

In order to get a maximum PAS signal, the acoustic wave generated from the modulated laser should form a standing wave inside the PA cell and the modulation frequency of the diode laser should match the resonant frequency of the PA cell. Therefore, the resonant frequency of the PA cell was measured with a CO concentration of 4.97%. The results of this measurement are shown in Figure 5. The data were fitted with a Lorentz contour. As can be seen in Figure 5, the resonant frequency of the PA cell was 1578.95 Hz. The full width at half maximum (FWHM) was 109.71 Hz. Therefore, the Q factor of such a PA cell was calculated to be 14.4.

**Figure 6:**
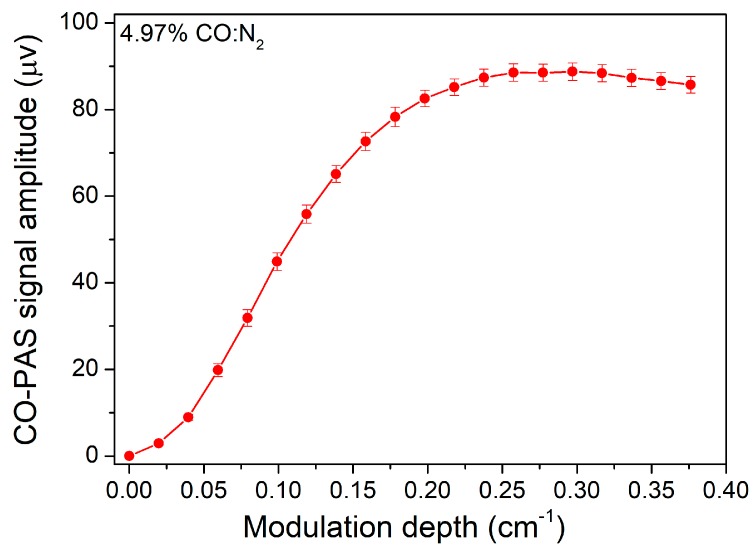
PAS signal amplitude as a function of modulation depth.

Besides the modulation frequency, the wavelength modulation depth should also be optimized to improve the PAS signal. The amplitude of the PAS signal as a function of the modulation depth was investigated experimentally and is shown in Figure 6. It can be seen that the PAS signal increased with an increase of the modulation depth at first, but when the modulation depth was larger than 0.3 cm^−1^, the PAS signal declined. Therefore, the optimum modulation depth was 0.3 cm^−1^, which agreed well with the theoretical calculation results shown in Figure 4.

**Figure 7:**
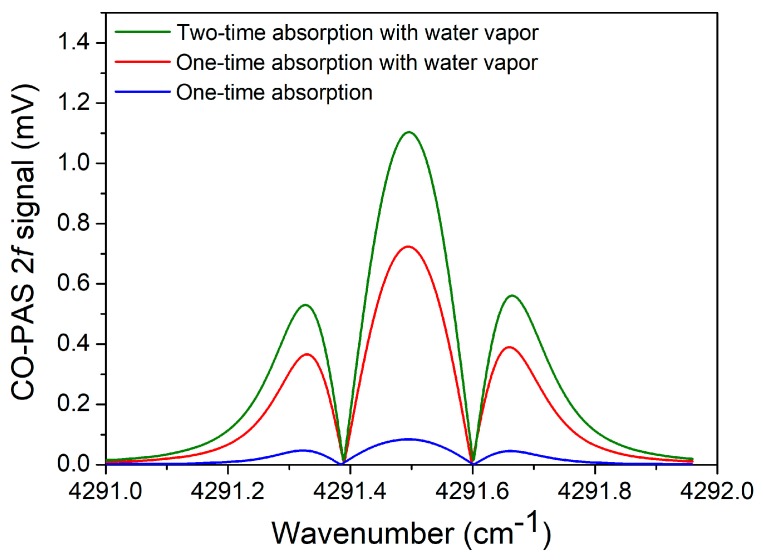
2*f* signal for CO-PAS detection with a modulation depth of 0.3 cm^−1^ consisting of a one-time absorption, two-times absorption, and water vapor, respectively.

For a gas molecule with a slow vibrational–translational (V–T) relaxation rate, an addition of other molecules, such as water vapor (H_2_O), can act as a catalyst for the V–T relaxation energy reactions efficiently. An enhancement of the CO-PAS signal was realized by the addition of water vapor to the CO/N_2_ gas mixture to improve the CO V–T relaxation rate. As shown in Figure 7, compared with the dry CO/N_2_ gas mixture, the addition of water vapor with a concentration of 1.24% resulted in an ~8 times signal enhancement. The 2*f* signal for the CO-PAS sensor was further investigated. When the right-angle prism was used to increase the absorption times, the amplitude of the 2*f* signal was 1.1 mV, which had a 1.52 times improvement compared with the signal of a single path. The improvement of 1.52 times was lower than 2 times in the ideal case, which was mainly due to the optical loss of the reflection and transmission.

**Figure 8:**
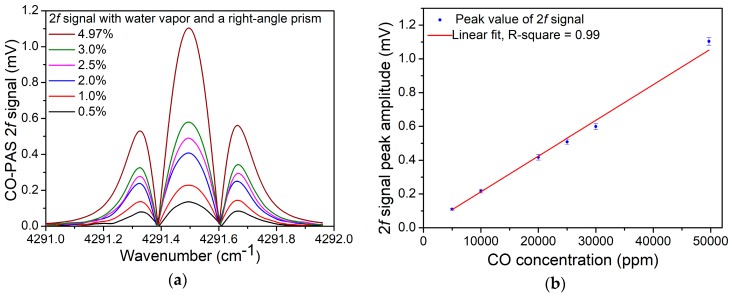
PAS signal with an optimum modulation depth of 0.297 cm^−1^. (**a**) 2*f* signal with different CO concentrations. (**b**) Signal peak value as a function of concentration.

The linear concentration response of the PAS-based CO sensor system was verified. The PAS 2*f* signal was detected with different concentrations of CO. A 4.97% CO/N_2_ mixture was diluted with pure N_2_. The measured results are shown in Figure 8a and the peak amplitude of the 2*f* signal as a function of CO concentration is shown in Figure 8b. Using a linear fitting, the R-square for the data shown in Figure 8b is ~0.99. Therefore, it could be concluded that the sensor system had an excellent linear response to the CO concentration levels. In order to measure the noise of such a sensor system, the PA cell was filled with ultra-high purity N_2_. The 1σ background noise was measured to 0.217 μV. Therefore, when the integration time was set to 1 s, the minimum detection limit (MDL) for the CO detection was 9.8 ppm.

**Figure 9:**
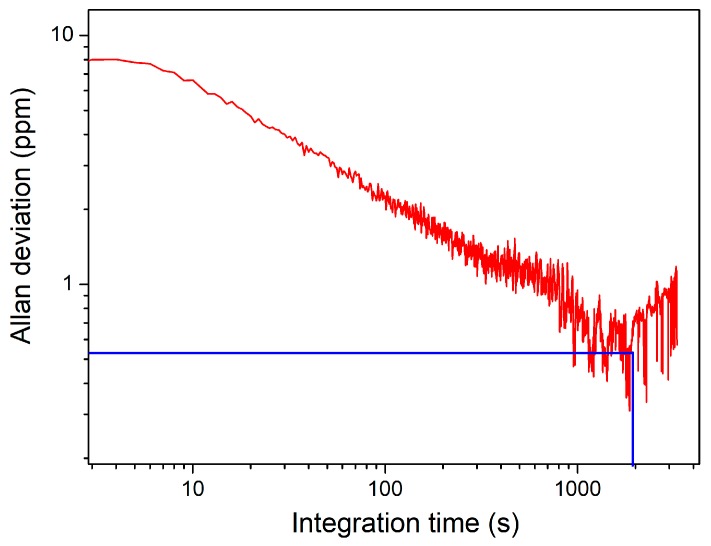
Allan deviation analysis for the PAS-based CO gas sensor.

The long-term stability of such a PAS-based sensor was evaluated with an Allan deviation analysis. The signal was measured for more than two hours with the resonant PA cell filled with pure N_2_. The results of measurements are shown in Figure 9. It showed that the PAS-based CO sensor system had excellent stability, and when the integration time of the lock-in amplifier was set to 1100 s, an MDL of 530 ppb for the detection of CO could be achieved.

## 5. Conclusions

In conclusion, a PAS-based CO gas sensor with a high optical power laser source and an enhanced absorption with double pass was demonstrated. A CW, DFB diode laser emitting at 2.3 μm with a high output power of ~8 mW was used as a strong excitation. A right-angle prism was used to reflect the laser beam parallelly inverted and pass through the PA cell twice to enhance the optical absorption. The WMS and second-harmonic detection techniques were used to reduce the noise of the sensor system. The modulation depth was optimized theoretically and experimentally to improve the PAS signal level. The experimental results agreed well with the calculated ones. Water vapor was added in the PAS sensor system to increase the V–T relaxation rate of the CO molecule, which resulted in an ~8 times signal enhancement compared with the using of a dry CO/N_2_ gas mixture. The linear response to the CO concentration was investigated, and the results showed that the reported PAS-based CO gas sensor has excellent performance characteristics. With an integration time of 1 s, an MDL of 9.8 ppm for CO detection was achieved. The long stability of such a sensor system was evaluated with the Allan deviation analysis. When the integration time was 1100 s, the MDL improved to be 530 ppb. The reported PAS-based CO sensor is useful in applications such as combustion processes and fire detection. Furthermore, the detection performance of the reported sensor can be improved in the future when a laser with a higher output power is available. If increasing optical absorption times or a right-angle prism made from materials with low absorption loss can be applied, the MDL of the reported CO-PAS sensor could be further improved.
